# Chronic Delivery of a Thrombospondin-1 Mimetic Decreases Skeletal Muscle Capillarity in Mice

**DOI:** 10.1371/journal.pone.0055953

**Published:** 2013-02-06

**Authors:** Gerald N. Audet, Daniel Fulks, Janelle C. Stricker, I. Mark Olfert

**Affiliations:** Division of Exercise Physiology, Center for Cardiovascular and Respiratory Sciences, West Virginia University School of Medicine, Morgantown, West Virginia, United States of America; Center for Cancer Research, National Cancer Institute, United States of America

## Abstract

Angiogenesis is an essential process for normal skeletal muscle function. There is a growing body of evidence suggesting that thrombospondin-1 (TSP-1), a potent antiangiogenic protein in tumorigenesis, is an important regulator of both physiological and pathological skeletal muscle angiogenesis. We tested the hypothesis that chronic exposure to a TSP-1 mimetic (ABT-510), which targets the CD36 TSP-1 receptor, would decrease skeletal muscle capillarity as well as alter the balance between positive and negative angiogenic proteins under basal conditions. Osmotic minipumps with either ABT-510 or vehicle (5% dextrose) were implanted subcutaneously in the subscapular region of C57/BL6 mice for 14 days. When compared to the vehicle treated mice, the ABT-510 group had a 20% decrease in capillarity in the superficial region of the gastrocnemius (GA), 11% decrease in the plantaris (PLT), and a 35% decrease in the soleus (SOL). ABT-510 also decreased muscle protein expression of vascular endothelial growth factor (VEGF) in both the GA (−140%) and SOL (−62%); however there was no change in VEGF in the PLT. Serum VEGF was not altered in ABT-510 treated animals. Endogenous TSP-1 protein expression in all muscles remained unaltered. Tunnel staining revealed no difference in muscle apoptosis between ABT-510 and vehicle treated groups. These data provide evidence that the anti-angiogenic effects of TSP-1 are mediated, at least in part, via the CD36 receptor. It also suggests that under physiologic conditions the TSP-1/CD36 axis plays a role in regulating basal skeletal muscle microvessel density.

## Introduction

Physiologic angiogenesis and homeostasis of adult blood vessels is a complex process that is highly regulated by a balance between positive and negative angiogenic proteins. While there is a substantial body of evidence on the role positive angiogenic factors, such as vascular endothelial growth factor (VEGF) [1], [2], [3], the role of negative angiogenic factors is incomplete and poorly understood [4].

There are a number of known angiogenic inhibitors, of which thrombospondin-1 (TSP-1) is thought to play a prominent role in skeletal muscle angiogenesis [4]. TSP-1 is a large (450 kD) extracellular protein that has a wide array of functions [5]. First discovered for its role in wound healing and platelet activation, it also has important roles in apoptosis, inflammation, nitric oxide signaling, and inhibition of positive angiogenic proteins [6], [7], [8], [9], [10]. Acting through CD36 (one of its receptors), TSP-1 has been shown to prevent endothelial cell adhesion, growth, and migration, as well as increase apoptosis [11], [12], [13]. The anti-angiogenic effects of TSP-1 in cancer pathology are well established. For example, a reduction in TSP-1 has been shown to increase tumor vessel growth, whereas pharmacological administration of several different TSP-1 mimetics has helped decrease tumor size and disease progression in animal models [14], [15], [16], [17].

Under physiologic conditions, TSP-1 expression has been shown to be responsive to exercise [18], [19], which suggests that TSP-1 may play a role in regulating exercise-induced skeletal muscle angiogenesis. TSP-1 KO mice have elevated skeletal muscle capillarity compared to wild-type mice, suggesting a critical role for TSP-1 in the physiological maintenance of capillaries [4]. Evidence in rats also shows that hindlimb unloading increases TSP-1 in association with decreases in skeletal muscle capillarity [3]. Recently, TSP-1 and it's receptor CD47 have also been suggested to play a pivotal role in skeletal muscle mitochondrial biogenesis, and therefore it is likely to be important in overall skeletal muscle function and adaptation to exercise stress [20].

Given the growing data implicating that importance of TSP-1 as a key angiogenic regulator in skeletal muscle, we sought to determine the consequences of chronically stimulating the TSP-1 pathway using ABT-510. ABT-510 is a mimetic of the conserved type I repeat region of TSP-1 which has been shown previously to inhibit angiogenesis through the CD36 receptor [12], [13], [21]. ABT-510 has been synthesized to have a longer half life in circulation than the native type I repeats of TSP-1 which allows for increased chronic stimulation of the TSP-1/CD36 pathway [22]. ABT-510 has been shown to be a potent inhibitor of vascular growth in tumorigenesis both *in vitro* and *in vivo* [15], [23], [24], [25], [26], [27], [28], which has been attributed to its binding to the CD36 receptor. Using this peptide we aimed to examine whether increased stimulation of the CD36 arm of the TSP-1 pathway affected skeletal muscle structure and function. Given that TSP-1 is a negative angiogenic regulator, we hypothesized that mice subjected to the ABT-510 would have lower skeletal muscle capillarity, mirrored by an altered balance between positive and negative angiogenic proteins.

## Methods

### Animals

This study used 10–12 week old male C57BL/6 mice purchased from Jackson Laboratories (Strain no. 000664, C57BL/6J, Bar Harbor, ME). Mice were randomly selected to be placed in two groups (control, n = 8; experimental n = 8). All procedures that involved animals were approved by the West Virginia University Institutional Animal Care and Use Committee.

### Exercise Testing Protocol

Whole body exercise capacity was assessed using a graded maximal exercise test (max test) 48 hours before pump implantation and then again 48 hours before muscle harvest (12 days after implantation). This was done to ensure that the exercise bout did not influence protein levels in muscles harvested on the final study day (Day 14) [18]. Maximal run test procedure: mice were given a 5 minute warm-up (speed <4 m/min) at a 10 degree incline on a commercially available rodent treadmill (Columbus Instruments, Columbus, OH). After the warm-up period, the treadmill was increased 2 m/min every 30 seconds until the animals reached exhaustion. Exhaustion was defined by a single observer as the inability of the animal to stay on the treadmill at a given speed, and/or more than 3 seconds spent on the shock grid. Animals were encouraged to run as fast and as long as possible by using forced air and a weak electrical deterrence. Final run time and velocity were recorded and compared between groups.

### Osmotic Pump Drug Delivery

The TSP-1 mimetic ABT-510 was generously provided by Abbott (Abbott, IL). This compound was chosen for use in this study based on previous and preliminary work showing its effectiveness in decreasing capillarity in tumors, both *in vitro* and *in vivo* [15], [22], [23], [24], [25], [29], [30]. ABT-510 was dissolved in 5% dextrose (vehicle) over a 48 hour period to ensure the compound was fully dissolved, and then inserted into mini-osmotic pumps (Model 1002, Alzet Osmotic Pumps, Cupertino, CA,). Pumps were filled with either the drug or vehicle solution 24 hours in advance of implantation as per manufacture instructions. We delivered the maximum concentration of 30 mg/kg/day based on the maximum pump capacity (100 µL), the mass of the animals, and the solubility of the drug (ABT-510 solubility curves [22]). Pumps were surgically inserted (flow-moderator first) subcutaneously in the scapular region while the mice were under anesthesia (2% isoflurane). The surgical site was disinfected with iodine and closed using sutures. Animals were then housed individually and observed daily for the remainder of the study for pain or distress. Pumps remained in the animals for 14 days, after which they were sacrificed and tissue/organ samples were collected and stored at −80° for later analysis.

### Morphometry

Hindlimb skeletal muscle was surgically excised and frozen in isopentane cooled by liquid nitrogen. Frozen tissue was cut using a −20°C cyromicrotome (Jung-Reichert Cryocut 1800: Cambridge Instruments, Germany) to yield 10 µm transverse sections. Great care was taken to ensure the cryosectioned muscles were cut along the transverse plane. Sections were stained for dipeptidly-peptidase IV (DPP IV) and alkaline phosphatase (AP) following the method of Lojda (1979), as applied to skeletal muscle tissue [31], [32]. A light microscope (Zeiss primo star, Zeiss, Oberkochen, Germany) was used to digitally acquire (Axiocam IC c3, Axiovision 4.8.2.0, Zeiss, Oberkochen, Germany) 20× images of the gastrocnemius, plantaris, and soleus. Capillary and myofiber counting was performed by a single individual blinded to group identification. For the gastrocnemius muscle, we obtained images in a checkerboard fashion across the entire muscle, thus both superficial and deep regions within the gastrocnemius could be included in the analysis. For the plantaris and soleus muscles, respectively, the entire muscle was imaged and analyzed. Counting was performed by visualization from acquired images using a custom program in MATLAB (version 7.0.0.27, The Mathworks, MA, USA) allowing the operator to visually mark and count the capillaries and fibers on each image. Capillary-to-fiber ratio (C∶F, number of capillaries/number muscle fibers), capillary density (CD, number of capillaries/mm^2^ muscle fiber area), and fiber cross sectional area (FCSA) were separately calculated for the gastrocnemius (GA), soleus (SOL), and plantaris (PLT) (n = 33–101 images/muscle/group).

### Protein Analysis

GA, SOL, and PLT muscles from each group were excised and flash frozen in liquid nitrogen. They were then separately homogenized in a lysis buffer containing 50 mM Tris/HCl (pH 7.4), 150 mM NaCl, 0.5% Triton X-100, and protease inhibitors (Complete Tablet, Roche Applied Science, Indianapolis, IN). Homogenates were centrifuged at 4°C, at 8,000 *g* for 10 minutes, and supernatants removed and placed in new tubes. Blood samples were obtained from the heart and allowed to coagulate on ice. They were then centrifuged at 3000 *g* for 10 minutes and flash frozen in liquid nitrogen. Total protein was measured by bradford assay (#23236 Pierce Coomassie Plus Protein Assay Kit, Thermo Scientific, Rockford, IL).

Quantification of VEGF was made from a total of 100 µg of protein using a commercially available ELISA kit according to the manufactures instructions (# MMV00, R&D Systems, Minneapolis, MN, USA). Quantification of VEGFR-2 and P-VEGFR-2 were made from a total of 100 µg of protein using a commercially available ELISA kit according to the manufactures instructions (# 7335S, #7340S, Cell Signaling, Danvers, MA, USA). TSP-1 and CD36 were analyzed via western blot. In brief, samples were separated on a 3–8% SDS-PAGE (NuPAGE Novex 3–8% Tris-Acetate Midi Gel, Invitrogen, Burlington, ON, Canada) and blotted onto a 0.45 µm nitrocellulose membrane (Pierce nitrocellulose membrane, Thermo Scientific, Rockford, IL). After blocking with 5% fat-free milk, membranes were probed using antibodies against TSP-1 (1∶1000, clone A6.1, #399300, Invitrogen, Burlington, ON, Canada), β-tubulin (1∶1000, #2148, Cell Signaling), CD36 (1∶250 #552544, BD Pharmingen, Franklin Lakes, NJ, USA), secondary HRP-conjugated anti-mouse (1∶1000, #p0260, Dako, GE Healthcare, Piscataway, NJ) and secondary HRP-conjugated anti-rabbit (1∶1000, #p0217, Dako). Proteins were visualized using chemiluminescence detection (Pierce ECL, Thermo Scientific, Rockford, IL) and digitally imaged (G∶BOX Gel imager, Syngene, Cambridge, UK) using Genesnap software (Ver. 7.01, Syngene, Cambridge, UK). Equal protein loading was verified by immunodetection of β-tubulin as our loading control. Quantification of protein expression levels were carried out using NIH Image J Software (v1.62) and expressed as densitometric arbitrary units (AU).

### Apoptosis

Nuclei exhibiting apoptotic changes were identified by TdT-mediated dUTP nick end labeling (TUNEL) according to the manufacturer's recommendations (Roche Molecular Biochemicals, Pleasanton, CA). Briefly, muscle cross sections were cut on a cryostat (10 µm) and fixed in 4% paraformaldehyde at room temperature, blocked in 3% H2O2 in 100% methanol at room temperature, and permeabilized in 0.1% Triton X and 0.1% sodium citrate. TUNEL reaction mix was added in a 1∶7.5 dilution, and the sections were incubated at 37°C for 1 h. Sections were reacted with fluorescein antibody for 30 min at 37°C, and substrate was added for color development. To control for false-positives, samples were then counter stained by DAPI staining to identify the nucleus. TUNEL staining was performed using a fluorescein TUNEL kit at 1∶7.5 dilution as recommended by the manufacturer (Roche Molecular Biochemicals, Pleasanton, CA). Positive nuclei were counted, and at high power (×400), it was determined whether they were associated with the myofiber or with the interstitial space. The number of positive nuclei is expressed per whole muscle section. Positive controls were created using a DNase treatment of 1 serial section (# AM1906, DNA-*free*, Ambion, Austin, TX). Negative controls were created by staining 1 serial section only for DAPI without TUNEL staining. This resulted in each sample containing 1 negative, 1 positive, and 2 experimental sections per slide.

### Statistics

All data are presented as means+/−SEM. To examine body mass, organ masses, muscle capillarity and molecular responses we used a student's T-test. A repeated measures ANOVA was used to analyze the maximal running test. An alpha level at *P*<0.05 was selected for statistical significance.

## Results

### Exercise Testing and Morphometry

#### Body and Muscle Mass

There was no significant difference in the absolute body or individual hindlimb muscle masses between ABT-510 (mimetic) and vehicle treated groups (Table 1). Heart and skeletal muscle normalized to body mass were not different between the groups (Table 1).

**Table 1:** Muscle and Body Masses.

	Vehicle	ABT-510
**Body Mass (g)**	27.5±1.23	27.1±0.87
**GA (mg)**	136.8±4.56	140.8±4.09
**PLT (mg)**	19.7±0.90	19.0±0.53
**SOL (mg)**	7.7±0.47	7.8±.048
**HRT (mg)**	123.5±5.76	134.3±5.82
**GA/BM (mg/g)**	5.0±0.07	5.2±0.12
**PLT/BM (mg/g)**	0.72±0.04	0.70±0.02
**SOL/BM (mg/g)**	0.29±0.01	0.28±0.01
**HRT/BM (mg/g)**	4.4±0.26	5.0±0.17

Values: Mean ± Standard Error.

* P = 0.05.

#### Maximal Running Test

There was no difference in maximal running speed between vehicle and mimetic treated animals pre- or post-treatment.

#### Morphometry

**Figure 1:**
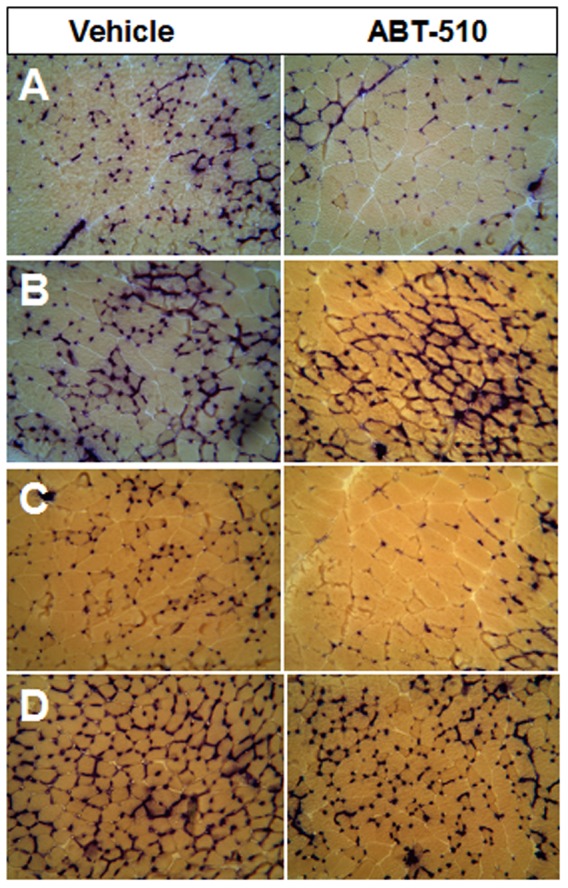
Representative figures of the histology sections. A) Superficial Gastrocnemius Muscle B) Deep Gastrocnemius Muscle C) Plantaris Muscle, D) Soleus Muscle.

**Figure 2:**
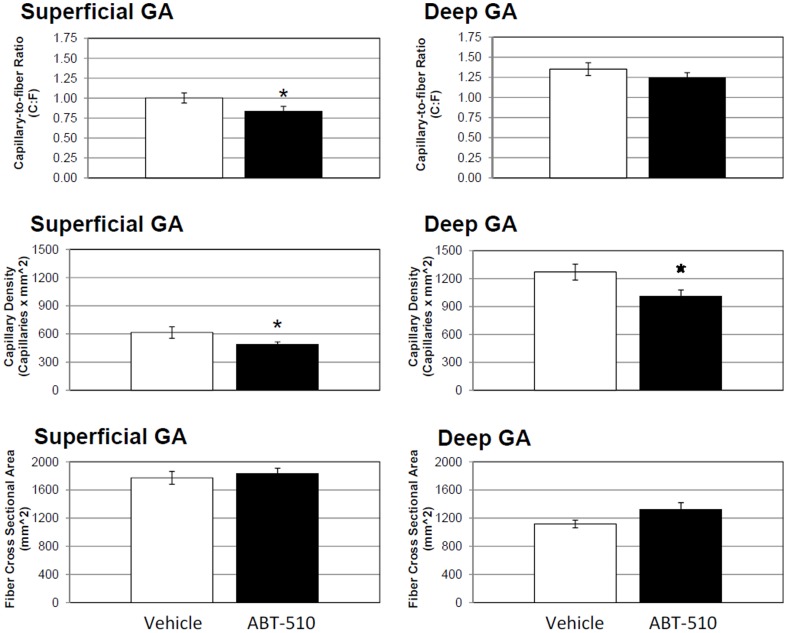
Superficial gastrocnemius capillarity, but not deep, is decreased in the ABT-510 group. Chronic administration of ABT-510 30 mg/kg/day decreased capillary to fiber ratio (C∶F) and capillary density (CD) in the superficial gastrocnemius (GA). Despite a similar trend, no significant changes in capillarity were seen in the deep GA. There was no change in fiber cross sectional area in either the deep or superficial portions of the GA. * = P≤0.05.

In the superficial region of the gastrocnemius (GA) muscle there was a 20% decrease in C∶F (P≤0.05) and a similar trend in CD (25% decrease, P = 0.055) in the mimetic group compared to vehicle (Figure 1 & 2). There was no statistical difference in C∶F of the deep portion of the GA between groups, however there was a significant 25% decrease in CD (P≤0.05). FCSA was not significantly different between groups in either portions of the GA.

**Figure 3:**
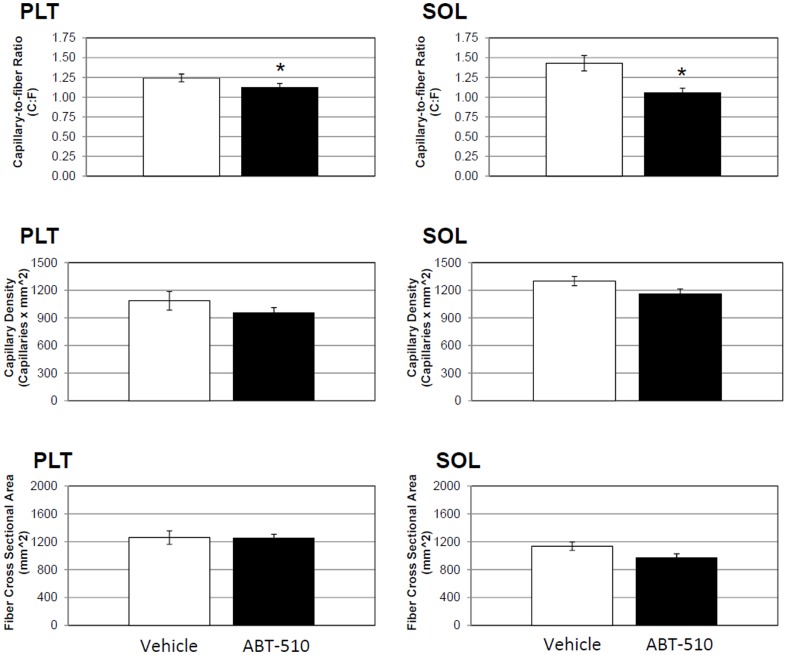
Plantaris and soleus capillarity is decreased in the ABT-510 group. Chronic administration of ABT-510 30 mg/kg/day decreased capillary to fiber ratio (C∶F) in the plantaris muscle (PLT) but did not decrease capillary density (CD). ABT-510 decreased capillary to fiber ratio (C∶F) and capillary density (CD) in the soleus (SOL). There was no change in fiber cross sectional area in either the PLT or SOL muscle. * = P≤0.05.

In the plantaris (PLT) muscle, there was a 11% decrease in the C∶F in the PLT in the mimetic group compared to vehicle (P≤0.05) (Figure 3). There was no difference in CD or FCSA in the PLT.

In the soleus (SOL) muscle, there was a 35% decrease in the C∶F in the SOL in the mimetic group compared to vehicle (P≤0.01) (Figure 3). There was no difference in CD (P = 0.08) or FCSA (P = 0.09).

### Endogenous VEGF, VEGFR-2, p-VEGFR-2, TSP-1, and CD36 Levels

**Figure 4:**
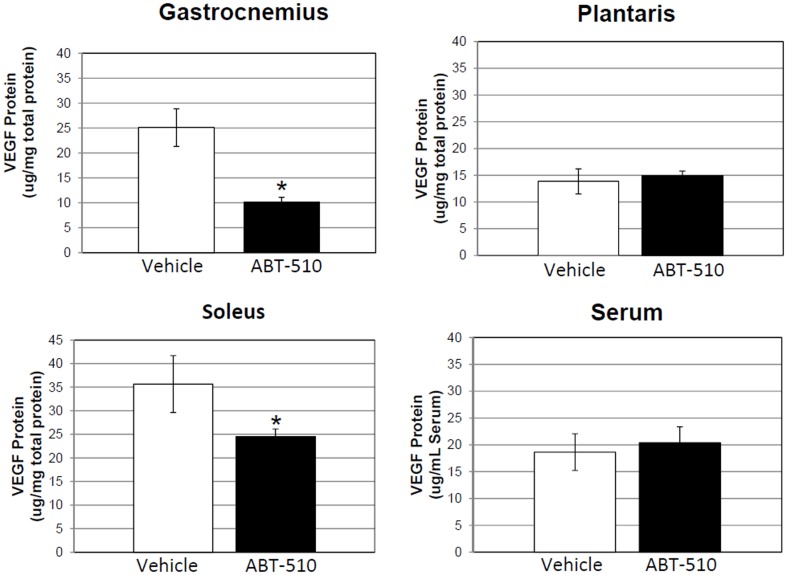
Skeletal muscle VEGF protein content is decreased in the ABT-510 group, but not serum levels. Chronic administration of the mimetic (ABT-510 30 mg/kg/day) decreased VEGF protein in the GA, and SOL muscles of C57/BLK6 mice. There was no change in the PLT muscle or in Serum levels. * = P≤0.05.

Skeletal muscle VEGF protein expression was decreased by 147% and 62% in the GA and SOL respectively (P≤0.05, Figure 4). In contrast, no significant change in VEGF protein expression was seen in the in the PLT. Serum VEGF levels were unchanged between ABT-510 and vehicle treated animals.

Total VEGFR-2 and phorphylated-VEGFR-2 (p-VEGFR-2) levels were also assessed in the GA. There was no difference in total VEGFR-2 levels, p-VEGFR-2 levels, or a ratio of VEGF-R2/p-VEGF-R2 between ABT-510 and vehicle treated mice (data not shown).

#### Endogenous TSP-1

protein expression of endogenous TSP-1 was not significantly different in any of the muscles analyzed (SOL, PLT, GA) between the treated groups (data not shown).

#### CD36 levels

There was no difference in the receptor levels of CD36 in the GA as assessed by western blot (data not shown).

### Apoptosis

**Figure 5:**
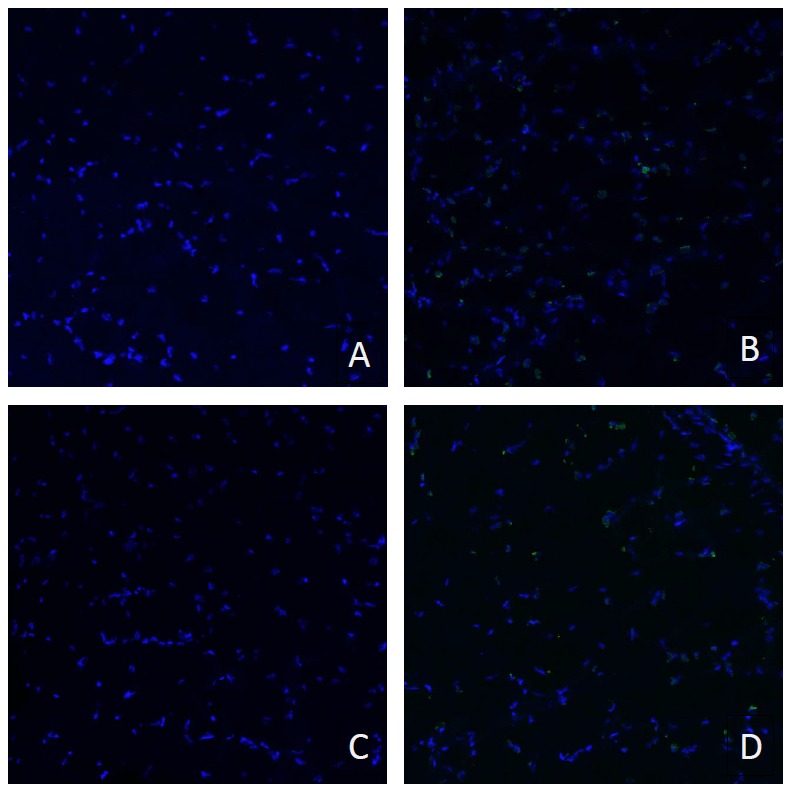
There is no difference in skeletal muscle apoptosis between the mimetic and control groups. TUNEL staining shows that chronic administration of the mimetic (ABT-510 30 mg/kg/day) did not increase or decrease skeletal muscle apoptosis in any of the muscles tested. Representative GA samples, PLT and SOL data not shown. A) Representative mimetic GA B) Representative positive control mimetic GA C) Representative control GA D) Representative positive control vehicle GA.

Apoptosis was assessed using TUNEL staining for fragmented DNA. There was no difference in the number of TUNEL positive nuclei in any of the muscles between the two treated groups (Figure 5).

## Discussion

The main finding of this study is that chronic exposure to the TSP-1 mimetic ABT-510 significantly decreases skeletal muscle capillarity (Figure 1). To our knowledge, these are the first data to show that chronic stimulation of the TSP-1/CD36 pathway in healthy mammals results in decreased skeletal muscle capillarity. These data are consistent with the anti-angiogenic function of TSP-1 and, more importantly, support the notion that TSP-1 through its CD36 receptor is a critical regulator of skeletal muscle capillarity under physiologic conditions. Indeed, when coupled with the previous observation that loss of TSP-1 increases skeletal muscle capillarity (25) these data provide evidence that the actions of TSP-1 directly influence homeostatic maintenance and/or development of skeletal muscle microvessels.

### Skeletal muscle capillarity is decreased in mimetic treated mice

TSP-1 has been shown previously to be important in maintaining skeletal muscle capillarity, where TSP-1 KO mice have approximately 2 fold the number of capillaries as WT controls [33]. Further, TSP-1 has been shown to be important in maintaining capillarity with hindlimb unloading [34]. In this manuscript we build upon this small body of evidence for the importance of TSP-1 in the context of the basal regulation of angiogenesis.

The actions of TSP-1 are complex and multifunctional, due in large part to the diversity of receptors it binds, such as lipoprotein receptor-related protein 1 (LRP-1), CD47, and CD36 [11], [35]. Our use of this the TSP-1 mimetic ABT-510, which is a potent CD36 binding peptide, resulted in a decrease in capillarity across three different distinct skeletal muscles. This suggests that TSP-1s anti-angiogenic properties in skeletal muscle involves a CD36 mediated mechanism. Indeed, this is consistent with the physiological activity of CD36 where TSP-1 binding to CD36 has been shown to be anti-proliferative, anti-angiogenic, and pro-apoptotic [12]. The importance of the TSP1/CD36 pathway has been proven in tumorigenesis, where down regulation of TSP-1 and its binding to CD36 results in a pro-tumorigenesis environment and increase in tumor size [14], [36], [37], [38]. Here we show similar actions in skeletal muscle under physiological conditions. This suggests a putative role for TSP-1 in decreasing vascularity across multiple tissues, and not just in tumors.

Given these findings, it is tempting to speculate that elevated skeletal muscle TSP-1 may serve as a biomarker for skeletal muscle dysfunction associated with several chronic conditions known to result in muscle capillary rarefaction, such as that found in diabetes [39], [40], and chronic heart and lung disease [41]. It may even be that therapeutic interventions to limit basal TSP-1 expression and/or reduce circulating levels of TSP-1 could be clinically exploited to attenuate the decrements in skeletal muscle function often accompanying these diseases.

### Chronic stimulation of the TSP1/CD36 pathway decreases VEGF

It has been shown previously that TSP-1 can counter the effects of VEGF by multiple mechanisms; including interacting directly with VEGF protein and disrupting its actions at the receptor level [42], [43], [44], [45], [46]. For example, TSP-1 has been suggested to bind VEGF via its type I repeat region (or 3TSR). Once bound the protein heterodimer is internalized by the scavenger receptor low density lipoprotein receptor-related protein 1 (LRP-1). Here, we show that a chronic administration of ABT-510 (a TSP-1 type I repeat mimetic), results in a decrease in total VEGF protein in both the gastrocnemius muscle and the soleus muscle, along with the decrease in capillarity. It is unclear why a similar decrease in VEGF protein was not seen in the PLT which also showed decreased capillarity, nevertheless these data support previous studies suggesting that TSP-1 may be binding and sequestering VEGF – resulting eventually in internalization and biological inactivation [42], [43], [44], [45], [46]. Explanations for the reduction in VEGF may also involve suppression of VEGF production and/or VEGF signaling. For example, there is a growing body of evidence showing that the internalization of the VEGF/VEGFR-2 complex by endocytosis results in downstream activation of several different signaling pathways [47]. There is also evidence that both CD36 and CD47 can associate with VEGFR-2, and when TSP-1 is present this association can prevent the VEGF ligand binding to its receptor, as well as inducing receptor dephosphorylation and preventing dimerization [13], [21], [48], [49]. In this way, TSP-1 could prevent activation and/or endocytosis of the VEGF receptor, blocking yet another biologically active arm of VEGF/VEGF-R complex. Regardless of the mechanism, our data supports the notion that TSP-1 likely plays a regulatory role involving VEGF, thereby enhancing its already potent inherent anti-angiogenic capacity.

In endothelial cells, TSP-1 can also disrupt VEGF actions at the receptor level by dephosphorylating the important receptor VEGFR-2 (Flk-1) [50]. TSP-1 can disrupt VEGFR-2 activation by binding the CLESH region of CD36, triggering a cascade of events which results in direct binding of VEGFR-2, and a resultant decrease in tyrosine phosphorylation of VEGFR-2 [12], [13], [21]. This includes dephosphorylating the important tyrosine 1175 region; which is critical to initiate downstream VEGF signaling. While it has also been shown previously that ABT-510 significantly decreases the amount and phosphorylation status of VEGFR-2 in endothelial cells *in vitro* [24], [51], *in vivo* we found no significant change in the phosphorylation levels of tyrosine 1175 of VEGFR-2. Given that the mimetic is a type I repeat, and that total VEGF protein levels were decreased, we were surprised that VEGFR-2 phosphorylation was also not decreased. But, because we only have one time point, it could be that levels of phosphorylation were lower earlier in the chronic dosage but by the end of the study returned to normal levels. While ABT-510 has been shown to decrease VEGFR-2 phosphorylation in tumors [24], it may be that this response is different in skeletal muscle under physiological conditions. It could also be that phosphorylation status *in vivo* is not lowered by endogenous TSP-1 (or the addition of a mimetic), rather TSP-1 limits or prevents an increase in phosphorylation levels with a VEGF stimulus. This is yet to be elucidated.

Administration of ABT-510 did not significantly alter endogenous levels of TSP-1 in skeletal muscle, indicating that there was no feedback response from the mimetic binding to CD36 to alter endogenous TSP-1 levels in skeletal muscle. However, the reduction of skeletal muscle VEGF protein levels in association with the mimetic adds support to the idea that physiologic angiogenesis is controlled by a balance between both positive and negative angiogenic factors, particularly VEGF and TSP-1. It would have been interesting to deliver a higher dose of ABT-510 (>30 ug/kg/day) which may have resulted in greater reductions in muscle VEGF and capillarity; however the dose used was the maximum we could deliver using the small pumps suitable for mice and the 2-week duration we sought. Although shorter duration would allow greater concentrations of drug delivery, we opted to deliver the drug for 14 days to ensure that vascular remodeling would have sufficient time to occur. It could be hypothesized that tipping the scale towards an anti-angiogenic state (stimulated by the presence of ABT-510) would result in a response of positive angiogenic factors to counter this challenge. However, at least with respect to VEGF this was not the case. A decrease in VEGF and no change in endogenous TSP-1 suggests that the muscle has been pushed towards an anti-angiogenic phenotype.

Regardless of mechanism, these data show that the altered angiogenic state of the skeletal muscle can be driven by an increase in a TSP-1 mimetic. The resulting decrease in capillarity establishes the importance of TSP-1/CD36 pathway in maintaining and regulating basal skeletal muscle microvessel density.

### Skeletal muscle and endothelial apoptotic levels do not differ between groups

In addition to its anti-angiogenic properties, TSP-1 is also been known to promote apoptosis [6], [52], [53], [54], [55], [56], [57], [58]. TSP-1 is known to induce apoptosis through the CD36 pathway, as shown in previous studies, including those using ABT-510 [5], [24], [59]. Endothelial apoptosis is a natural mechanism by which capillary regression occurs [60], [61], [62], [63], and has been shown to be central in muscle atrophy during some pathologies, aging, and disuse [64], . Knowing this, we performed TUNEL staining to look at end stage apoptosis (DNA fragmentation) in all three of the excised muscles. Surprisingly, we found no evidence of apoptosis in any of the muscles, either myocyte or endothelial. The reason for this remains unclear. This is, however, the first *in vivo* report of ABT-510s affects on skeletal muscle and it could be that ABT-510 is not sufficient to induce myocyte apoptosis in skeletal muscle under these conditions. Indeed, much of the work with TSP-1 and apoptosis pertains to cancer models (e.g. tumorigenesis), endothelial cells, and is typically done *in vitro*. It is possible that TSP-1 is not as potent a pro-apoptotic protein in skeletal muscle as that seen in endothelial cells or tumors. This is supported by the finding that there was no change in muscle fiber cross-sectional area (Figures 2 and 3). Whether or not the ABT-510, or the TSP-1/CD36 axis, alters *in vivo* skeletal muscle myocyte apoptosis will require further study.

However, this does not explain why we saw no endothelial cell apoptosis. As mentioned previously, our study only examines one time point. It is possible that apoptosis occurred at an earlier time point than our assessment at the 14 day time point. Indeed, it has been shown previously that hindlimb unloading results in capillary loss in as little as 5 days [34]. If changes in endothelial cell number, and therefore total vasculature, are occurring by 5–7 days it may not be surprising that evidence of apoptosis has been lost by the end of the study (14 days). That is, perhaps apoptosis has already occurred, the cells cleared from the tissue, and the architecture of the vascular network at 14 days has already returned to a new steady state condition absent of apoptotic nuclei. We have yet to elucidate this and more experimentation is needed to determine exactly when apoptosis and regression is occurring.

### Summary

In conclusion, we provide evidence for the importance of TSP-1/CD36 pathway in regulating basal skeletal muscle capillarity by showing that a chronic dosage of a TSP-1 mimetic for the CD36 pathway decreases skeletal muscle capillarity and VEGF expression. We did not find any differences in the VEGFR-2 expression or its phosphorylation status, nor was there greater skeletal muscle apoptosis. These data show that, despite the multifunctional effects of TSP-1 and its CD36 receptor, the primary consequence of elevating circulating TSP-1 (i.e. using a TSP-1 mimetic targeted to its CD36 receptor) relates to its anti-angiogenic function. These data may be useful in exploring therapeutic interventions for individuals with skeletal muscle dysfunction resulting from capillary regression.
